# Continuing to Advance Epidemiology

**DOI:** 10.3389/fepid.2021.782374

**Published:** 2021-11-12

**Authors:** Tobias Kurth

**Affiliations:** Institute of Public Health, Charité - Universitätsmedizin Berlin, Berlin, Germany

**Keywords:** epidemiology, causal inference, prediction, education, COVID-19, early career researcher (ECR), population health, public health

## Introduction

Historically, successes in improving human health have been rooted in diligent observation and careful analysis of the given situation and data. In light of new, solid evidence contradicting prevalent accepted beliefs, the field of epidemiology must remain dynamic and be open to new ideas that further evolve its growth and ultimately improve health. Pioneering work in epidemiology includes the systematic investigation into the origins of the cholera outbreak in London by John Snow in the 1800s. He observed that the spread of the disease was linked to communal drinking water sources. This careful observation ultimately led to the understanding that contaminated water contained the causal agent of the disease, challenging the contemporarily accepted miasma theory (1). Such systematic investigations to identify disease risk factors, modes of transmission, and consequences remain essential today and have substantial implications for population health. For instance, the early recognition that SARS-CoV2 is primarily transmitted through aerosols was quintessential to inform public health policy and decision-making during the ongoing pandemic (2). Through objective observation and analysis, the development and implementation of appropriate interventions can be conducted in a reasonable time frame.

## What is Epidemiology?

Epidemiology is one of the mainstays of medicine and public health. It is a rapidly evolving field that lies at the intersection between clinical and population-based research. The field concerns itself with topics ranging from field observation and data collection to study design and analysis and pursues the overarching goals of furthering scientific discourse, informing decision-making, and improving health on the population level. Furthermore, as epidemiology continues to evolve and expand its scope, it is constantly being redefined. A review by Frérot and colleagues of published and gray literature from the last decades identified 102 unique definitions of “epidemiology,” with the majority containing the words “population,” “study,” “disease,” “health,” or “distribution” (3). Over time, they found an increasingly prevalent use of the terms “control” and “health” and a diminishing emphasis on “disease,” indicating the dynamic of the field and shifting to new focuses (3). Especially in the last 2 years, the field has experienced drastic change and growth, mainly in response to the new challenges that continue to arise during the ongoing Covid-19 pandemic and the need for innovative solutions.

## Current Challenges in Epidemiology

While widely regarded as a science that provides numbers of existing disease stages and their occurrences—which are key components that, when applied correctly, have been very useful throughout the Covid-19 pandemic—epidemiology, as a field, does more than just observe and quantify. In particular, in addition to answering descriptive questions, many epidemiological studies address prediction aims, while others seek to study the causes and effects of health stages. Correctly designing studies and applying proper data analysis methods to answer these questions are crucial components of modern epidemiology (4). Indeed, only through rigorous study conduct and application of methods can we expect to further our understanding and ultimately improve health on the population level.

The Covid-19-pandemic demonstrated that systematically collected data need to rapidly be made accessible to researchers around the globe so that they can be adequately analyzed to inform a proper response in a timely manner. However, the quality of many Covid-19 publications left much to be desired, underscoring the importance of proper use of research methodology and the impact of inaccurate conclusions (5–8). The Covid-19 pandemic has also led to a massive overload of the publishing system in epidemiology and other biomedical fields by a substantial increase in the number of submitted papers, with a concurrent lack of time for scientists to conduct proper peer review in an acceptable time frame (9).

In recent years, epidemiology has been confronted with new data collection methods leading to massive, ever-expanding databases, often referred to as big data, and automated algorithms to analyze them (e.g., machine learning). However, when addressing questions on adequate prediction and cause and effects, understanding and applying the proper analytic framework is a fundamental necessity (10). The increasing availability of data alone will not help improve population health if we only observe them without interpreting the underlying reality (10). New, innovative concepts are being developed, mainly in the intersection between predictive and causal frameworks (11–13) that will help to improve our understanding and provide advances.

We have observed a substantial lack of preparedness during the Covid-19 pandemic that should be addressed in revising education of related fields, such as medicine, public health, health communication, medical informatics, psychology, and others, with core modern epidemiological content. For timely and meaningful preparedness, we also need to extend international and interdisciplinary collaboration as well as improved fact-based communication to the public and decision-makers. The latter includes making scientific content open access with all its components. Lastly, we need to provide adequate opportunities for early career researchers to shape the future of epidemiology.

In the following sections, we will summarize pressing challenges currently facing the field of epidemiology, related to choosing the right framework and applying proper methodologies, in education and the promotion of interdisciplinary collaboration, and in improving health communication, encouraging open science, and building transparent peer review structures. Finally, we will discuss the challenges of engaging early career researchers and incorporating them into all aspects of the editorial work. Challenges for the specific subareas of epidemiology will be discussed in the respective sections of the journal.

## Choosing the Proper Framework: Description, Prediction, and Causal Inference

In many traditional epidemiology and biomedical circles, a statement about a causal relationship is considered justified if and only if it is the result of a randomized controlled trial. This implies that in all observational studies, which comprise the vast majority of the published literature, it is only possible to report and draw conclusions about loosely defined associations, which have unclear implications in terms of interventions and policy recommendations (14). While well-conducted randomized experiments are justifiably the gold-standard in biomedical research, they cannot, due to ethical and practical constraints, provide answers to many of the pressing questions in public health and adjacent domains. For this reason, the continued development and implementation of rigorous methodological approaches to provide answers to important causal questions is essential for the field.

The “causal revolution” at the end of the twentieth century represented a pivotal point in observational research and progress in the field of epidemiology. The introduction of directed acyclic graphs (and the underlying mathematical theory) by Pearl, Robins, and others (14–16) revolutionized how observational data can be used to answer causal questions, and new concepts are continuously being developed (4). In addition, the implementation of rigorous, modern methodological approaches, such as Robin's generalized methods (g methods) (17) and the target trial emulation framework (18, 19), have substantially improved research of causes and effects. However, there remains substantial room for improvement in the understanding and application of these methods. They have also improved our understanding that the correct target population needs to be selected when conducting clinic- or population-based studies (20).

Confronted with the causal revolution and the growing field of data science, there emerged an urgent need for clarity in delineating the main tasks underlying epidemiological research: description, prediction, and counterfactual prediction (or causality) (4). Each of these three overarching tasks has a unique set of characteristics, methodology, and challenges. The common confusion between these three frameworks (e.g., applying methods of prediction to answer a causal question) often leads to fundamental mistakes. Several examples of such problems were observed during the Covid-19 pandemic, during which inappropriate applications of methods led to the interpretation of non-causal associations as causal effect estimates (6, 8). Instead of banning specific phrases, as some biomedical journals have done, it is pertinent to encourage explicit statements of clearly operationalized study aims to reduce ambiguity and assess whether the methods used are actually suitable (21). Thus, *Frontiers in Epidemiology* will focus on the appropriate choice and application of the specific framework to answer specific research questions of description, prediction, and causal nature in an appropriate target population to improve the accuracy and interpretability of scientific findings. For example, we encourage authors of observational studies addressing causal questions to use causal language to describe their aims and to submit the directed acyclic graph used to inform their variable selection strategies, design, and analysis choices.

## Getting the Word Out: Education, Transparency, and Dissemination

A second set of challenges centers on the spreading and exchange of knowledge. High-quality education is an essential component of fostering scientific dialogues. We must promote the foundational, structured training of the next generation of epidemiologists and provide opportunities for continued learning to stay up-to-date and for interdisciplinary exchange. Though strongly rooted in historical tradition, it is important that we do not lose sight of the fact that epidemiology, as a field, is rapidly evolving. In this light, we need to push for the integration and continued updating of key conceptions of modern methodology in the curricula of epidemiology, public health, and biomedical training programs. The lack of proper application of methods and transparent reporting in many clinical and biomedical research studies reminds us that researchers have a societal responsibility when conducting and reporting scientific studies (7). Thus, one aim of *Frontiers in Epidemiology* is to regularly invite papers that showcase cutting-edge epidemiological methods and best practice application examples, as well as those which describe common biases and propose innovative design and analytic strategies to avoid them. The journal also encourages the submission of papers challenging current concepts or expanding upon them, and will actively promote training and advances in peer review.

To further scientific discourse and ensure a timely discussion of relevant topics, it is essential that scientific communication is openly accessible to the research community at-large as well as to the public. Furthermore, all aspects of peer review should be credited and openly reported for full transparency and to promote scientific integrity. Though it became glaringly apparent during the Covid-19 pandemic, a lack of direct access to original research or commentaries also hinders scientific progress in non-pandemic times. Without it, essential scientific interaction is restricted, as such information is needed for researchers to initiate or modify projects and decision-makers to implement necessary preventative measures. Like all journals within the *Frontiers* family, *Frontiers in Epidemiology* is an open-access journal with an open peer-review process. We further explicitly encourage authors to pre-register or publish research protocols and make their analysis code and, when possible, their research data publicly available.

Informing the public about the research of health aspects is an essential societal service. Epidemiology plays an essential role in communicating relevant measures of health conditions and justifications for findings in light of what is already known. Once again, we learned lessons during the Covid-19 pandemic about the confusion that inaccurate or incomplete communication of disease prevalence and incidence or associated consequences of a disease can create (22). Health communication starts with well-presented original research that should discuss aspects of the potential consequences on the individual and population level. One of the primary roles of a journal is to openly disseminate scientific information to the research community, decision-makers, and the public. Therefore, *Frontiers in Epidemiology* will assist in transferring and distributing knowledge and provide summarizing commentaries to the public and decision-makers.

## Thinking Outside of the Box: Promoting Interdisciplinary, Intersectionality, and New Voices

While the lack of early involvement of methodologists in biomedical research projects is known, at least to methodologists (7), epidemiologists should seek out opportunities to exchange and collaborate with scientists from other fields to expand their horizons and find innovative ways to improve their scientific practice. For example, during the Covid-19 pandemic, there were intensive exchanges between the fields of epidemiology, physics, mathematics, economics, and computer science. We should continue with this momentum and explore possibilities for collaboration with other domains such as engineering, law, and art. *Thus, Frontiers in Epidemiology* will specifically invite papers from experts of other fields for their perspectives on the intersections of their fields with epidemiology.

A central tenet of the scientific process is to challenge what we already know. In light of new evidence, we must be willing to reconsider our established beliefs and accepted knowledge. Taking an intersectional approach in our research brings additional complexity, but better reflects the reality of the world around us; a challenge that we must rise to meet in the next stage of evolution of our field. Instead of investigating individual determinants of health in a vacuum, modern research should explicitly aim to be inclusive and to address diversity with intention. Since the challenge of integrating adequate intersectional perspectives applies to all subsections of epidemiological research, it is essential to develop innovative methodological approaches to incorporate these aspects into research (23).

Scientific fields grow from high-level discussions and discourse. This is also true for epidemiology. Such discussions are often triggered by early career researchers who bring in new energy and ideas, which challenge existing theories and are essential to further improve the field. Early career researchers should not be met with resistance when they challenge legacy frameworks and accepted analytical approaches, but rather, we need to create a space in which they, too, can engage in open dialogue with more established researchers in the field. Early career researchers are an engaged group also eager to help improve the field in terms of important professional aspects, such as mental health, work-life balance and reverse mentoring (24, 25). *Frontiers in Epidemiology* will actively seek to promote the voices of early career researchers and provide a platform to foster innovative thinking and the exchange of ideas. We invite them to be active across all levels of the journal; as authors, peer reviewers, and editors. We explicitly encourage them to submit research papers challenging accepted narratives with convincing arguments, thereby generating new perspectives and motivating us to think about long-standing problems in new ways. In cooperation with the *Frontiers* publishing group, we pledge to work on concepts that improve the training and involvement of early career researchers in the journal's activities.

## Conclusion

*Frontiers in Epidemiology* aims to provide a platform for tackling the above-mentioned grand challenges of the field with innovative research, methods-focused exchange, and critical thinking. Each specialty section of the journal will address crucial research areas that affect how epidemiology can improve health in specific populations and stimulate critical exchange about the current narratives. Across the specialty sections, the journal encourages the implementation of modern views of epidemiology and its development, focusing on early career researchers.

## Author Contributions

The author confirms being the sole contributor of this work and has approved it for publication.

## Conflict of Interest

Outside the content of this paper, the author declares having received honoraria from Eli Lilly & Company and TotalEnergies for providing methodological advice, from Teva for participating in a symposium, and from The BMJ for editorial services.

## Publisher's Note

All claims expressed in this article are solely those of the authors and do not necessarily represent those of their affiliated organizations, or those of the publisher, the editors and the reviewers. Any product that may be evaluated in this article, or claim that may be made by its manufacturer, is not guaranteed or endorsed by the publisher.

## References

[1] Snow J. On the Mode of Communication of Cholera. (1849) Available online at: https://collections.nlm.nih.gov/ext/cholera/PDF/0050707.pdf

[2] Greenhalgh T Jimenez JL Prather KA Tufekci Z Fisman D Schooley R. Ten scientific reasons in support of airborne transmission of SARS-CoV-2. Lancet. (2021) 397:1603–5. 10.1016/S0140-6736(21)00869-2 33865497 PMC8049599

[3] Frérot M Lefebvre A Aho S Callier P Astruc K Aho Glélé LS. What is epidemiology? changing definitions of epidemiology 1978–2017. PLoS ONE. (2018) 13:e0208442. 10.1371/journal.pone.0208442 30532230 PMC6287859

[4] Hernán MA Hsu J Healy B. A Second chance to get causal inference right: a classification of data science tasks. Chance. (2019) 32:42–9. 10.1080/09332480.2019.1579578

[5] Wynants L Van Calster B Collins GS Riley RD Heinze G Schuit E. Prediction models for diagnosis and prognosis of covid-19 infection: systematic review and critical appraisal. BMJ. (2020) 369:m1328. 10.1101/2020.03.24.20041020 32265220 PMC7222643

[6] Griffith GJ Morris TT Tudball MJ Herbert A Mancano G Pike L. Collider bias undermines our understanding of COVID-19 disease risk and severity. Nat Commun. (2020) 11:5749. 10.1038/s41467-020-19478-2 33184277 PMC7665028

[7] Van Calster B Wynants L Riley RD van Smeden M Collins GS. Methodology over metrics: current scientific standards are a disservice to patients and society. J Clin Epidemiol. (2021) 5:18. 10.1016/j.jclinepi.2021.05.018 34077797 PMC8795888

[8] Westreich D Edwards JK van Smeden M Comment on Williamson. The table 2 fallacy in a study of COVID-19 mortality risk factors. Epidemiology. (2021) 32:e1–2. 10.1097/EDE.0000000000001259 33065610

[9] Kurth T Piccininni M Loder EW Rohmann JL. A parallel pandemic: the crush of covid-19 publications tests the capacity of scientific publishing. BMJ Opinion. (2020) Available Online at: https://blogs.bmj.com/bmj/2020/05/26/a-parallel-pandemic-the-crush-of-covid-19-publications-tests-the-capacity-of-scientific-publishing/ (accessed January 21, 2021).

[10] Pearl J. The seven tools of causal inference, with reflections on machine learning. Commun ACM. (2019) 62:54–60. 10.1145/3241036

[11] Sperrin M Martin GP Pate A Van Staa T Peek N Buchan I. Using marginal structural models to adjust for treatment drop-in when developing clinical prediction models. Stat Med. (2018) 37:4142–54. 10.1002/sim.7913 30073700 PMC6282523

[12] Piccininni M Konigorski S Rohmann JL Kurth T. Directed acyclic graphs and causal thinking in clinical risk prediction modeling. BMC Med Res Methodol. (2020) 20:179. 10.1186/s12874-020-01058-z 32615926 PMC7331263

[13] Lin L Sperrin M Jenkins DA Martin GP Peek N. A scoping review of causal methods enabling predictions under hypothetical interventions. Diagn Progn Res. (2021) 5:3. 10.1186/s41512-021-00092-9 33536082 PMC7860039

[14] Pearl J Mackenzie D. The Book of Why: The New Science of Cause and Effect. 1st ed Basic Books. (2018).

[15] Robins J. A graphical approach to the identification and estimation of causal parameters in mortality studies with sustained exposure periods. J Chronic Dis. (1987) 40:139S−161S. 10.1016/S0021-9681(87)80018-8 3667861

[16] Pearl J. Causal diagrams for empirical research. Biometrika. (1995) 82:669–88. 10.1093/biomet/82.4.669

[17] Hernán MA Robins JM. Causal Inference: What If. London: Chapman & Hall/CRC. (2020).

[18] Hernán MA Robins JM. Using big data to emulate a target trial when a randomized trial is not available. Am J Epidemiol. (2016) 183:758–64. 10.1093/aje/kwv254 26994063 PMC4832051

[19] García-Albéniz X Hsu J Hernán MA. The value of explicitly emulating a target trial when using real world evidence: an application to colorectal cancer screening. Eur J Epidemiol. (2017) 32:495–500. 10.1007/s10654-017-0287-2 28748498 PMC5759953

[20] Dahabreh IJ Haneuse SJ-PA Robins JM Robertson SE Buchanan AL Stuart EA. Study designs for extending causal inferences from a randomized trial to a target population. Am J Epidemiol. (2021) 190:1632–42. 10.1093/aje/kwaa270 33324969 PMC8536837

[21] Hernán MA. The C-Word: scientific euphemisms do not improve causal inference from observational data. Am J Public Health. (2018) 108:616–9. 10.2105/AJPH.2018.304337 29565659 PMC5888052

[22] Tosi D Verde A Verde M. Clarification of misleading perceptions of COVID-19 fatality and testing rates in italy: data analysis. J Med Internet Res. (2020) 22:e19825. 10.1136/jech-2020-22e19825 32490842 PMC7301630

[23] Mandelbaum J. Advancing health equity by integrating intersectionality into epidemiological research: applications and challenges. J Epidemiol Commun Health. (2020) 74:761–2. 10.1136/jech-2020-213847 32527861

[24] Holman C Weissgerber T Brasanac J Oliveira M Holst M Drude N. Mental health of early career researchers: COVID-19 brings the community together (virtually). (2021) Available online at: https://ecrlife.org/mental-health-of-early-career-researchers-covid-19-brings-the-community-virtually-together/ (accessed September 19, 2021).

[25] Raza A Onyesoh K. Reverse mentoring for senior NHS leaders: a new type of relationship. Future Healthc J. (2020) 7:94–6. 10.7861/fhj.2019-0028 32104775 PMC7032576

